# Dose–Dependence Relationship Between Radiotherapy and Oral Mucositis in Patients With Head and Neck Cancer: A Retrospective Study

**DOI:** 10.1155/ijod/3972232

**Published:** 2026-04-03

**Authors:** Samuel Rocha França, Flávia Carvalho Pinto, Victor Bento Oliveira, Katlyn Djéssi Silva Andrade, Priscilla Parente Viana, Filipe Nobre Chaves, Denise Hélen Imaculada Pereira de Oliveira, Marcelo Bonifácio da Silva Sampieri

**Affiliations:** ^1^ School of Dentistry, Sobral Campus, Universidade Federal do Ceará, Sobral, Ceará, Brazil, ufc.br; ^2^ School of Dentistry, Oral Pathology and Oral Surgery, Periodontology Division, Universidade Federal of Minas Gerais, Department of Dental Clinics, Belo Horizonte, Minas Gerais, Brazil

**Keywords:** epidemiology, head and neck cancer, observational study, oral mucositis, radiotherapy

## Abstract

**Introduction and Purpose:**

To evaluate the association between radiotherapy (RT) exposure and the presence and severity of radiation‐induced oral mucositis (RIOM) in patients with head and neck cancer (HNC).

**Methods:**

This retrospective observational study analyzed medical records of 121 patients with HNC treated with 3D conformal RT at a referral hospital in Brazil (2020–2023). Sociodemographic, clinical, and RT‐related variables were collected. RIOM was clinically assessed using World Health Organization (WHO) criteria and analyzed using three approaches: presence of RIOM, overall severity (grades 0–4), and severity among affected patients (grades 1–4). Analyses included descriptive, bivariate, and adjusted multivariable binary and ordinal logistic regression models.

**Results:**

RIOM was identified in 51 patients (42.1%). No cases of WHO grade 4 were observed. In multivariable analysis, intraoral tumor (aOR = 9.06; 95% CI: 3.51–23.38; *p* < 0.001), number of RT sessions (aOR = 1.11 per session; 95% CI: 1.04–1.17; *p*  < 0.001), cumulative dose (aOR = 1.05 per Gy; 95% CI: 1.02–1.08; *p* = 0.001) and female sex (aOR = 3.10; 95% CI: 1.13–8.43; *p* = 0.027) were independently associated with RIOM presence. Intraoral tumor and increasing RT exposure remained significantly associated with greater RIOM severity (*p*  < 0.01) in ordinal models. Preventive laser therapy showed a protective trend in bivariate analysis but did not remain significant after adjustment.

**Conclusion:**

RT exposure and intraoral tumor location were the main factors associated with both the presence and severity of RIOM, highlighting the importance of anatomical site and radiation burden for risk stratification.

## 1. Introduction

Head and neck cancer (HNC) accounted for ~758.000 new cases worldwide in 2022, with squamous cell carcinoma as the predominant histological type. Cancers of the oral cavity represent a substantial proportion of this burden and occur more frequently in men. In Latin America, including Brazil, socioeconomic inequalities contribute to delayed diagnosis and higher mortality, with Brazil showing particularly high incidence of oral cavity and tongue cancers. Tobacco and alcohol remain major risk factors, alongside emerging increases in HPV‐associated oropharyngeal cancer in urban populations and lip cancer related to chronic solar exposure in rural areas [1].

Management of HNC commonly involves radiotherapy (RT), which, despite its therapeutic role, frequently damages healthy oral tissues, particularly the salivary glands and oral mucosa [2, 3]. Radiation‐induced oral mucositis (RIOM) is a major complication, characterized by pain, dysphagia, and increased susceptibility to oral infections, and represents a key determinant of impaired oral health–related quality of life [4]. Salivary hypofunction and xerostomia further exacerbate functional and psychosocial burden during and after treatment [3, 4].

Identifying clinical and biological predictors of RIOM is essential to stratify patients at higher risk and guide preventive and curative strategies. Evidence indicates that factors such as treatment protocols, oncological diagnosis, and genetic susceptibility influence the occurrence and severity of RIOM [5–7]. Early recognition of these predictors may allow timely interventions, including photobiomodulation (low‐level laser therapy), thereby reducing morbidity and minimizing interruptions in antineoplastic therapy, which are critical for tumor prognosis and oncologic outcomes [8, 9].

Therefore, this retrospective study aimed to evaluate the prevalence and severity of RIOM and its dose‐dependent relationship with RT exposure in HNC patients, providing clinical insights into this condition.

## 2. Methods

### 2.1. Study Design and Setting

This retrospective observational study was reported in accordance with the STROBE guidelines [10]. Data were collected from medical records of patients diagnosed with HNC who underwent RT at Santa Casa de Misericórdia de Sobral (SCMS), a referral hospital in northeastern Brazil, between January 2020 and December 2023. The study was conducted in accordance with the principles of the Declaration of Helsinki. Approval was obtained from the Institutional Research Ethics Committee of Universidade Estadual Vale do Acaraú (No. 5.530.751). Informed consent was waived given the study’s retrospective design and the exclusive use of secondary data from medical records.

### 2.2. Participants and Eligibility

A consecutive sampling strategy was employed, identifying all medical records of HNC patients treated at SCMS during the study period.

Eligible records were required to document: (1) confirmed histopathological diagnosis of malignant neoplasia of the head and neck region; (2) treatment with external beam RT, either completed or ongoing at the time of data collection; and (3) availability of complete clinical records detailing RT sessions and systematic assessment of oral mucositis.

Exclusion criteria were: (1) patients receiving concomitant chemotherapy, to avoid confounding toxicities; (2) records with missing data on RIOM status, number of RT sessions T or dosimetry; and (3) patients undergoing palliative RT, due to differences in fractionation and intent of care. No age restrictions were applied.

### 2.3. Data Collection

All data were extracted retrospectively by trained investigators (S.M.R.F., F.C.P., K.D.S.A., and P.P.V.) using a standardized spreadsheet to ensure consistency across the sample. Variables were retrieved from institutional medical, dental, and RT documentation routinely used in hospital practice.

Demographic data included sex (female/male) and age (years) at the time of diagnosis. Clinical information encompassed the tumor location (dichotomized as intraoral or extraoral) and the histopathological type (squamous cell carcinoma vs. others). Preventive laser therapy was recorded as a binary variable (yes/no), based on documentation of its use at least once during any RT session prior to the detection of RIOM.

RT exposure was assessed using two continuous measures: total number of sessions and cumulative dose (Gy) at data collection, and corresponding values at RIOM documentation among patients with WHO grades 1–3.

### 2.4. Outcomes

The primary outcome was the presence of RIOM, clinically diagnosed and graded by specialized stomatologists according to the World Health Organization (WHO) criteria [11]. For analysis, RIOM was stratified into three approaches to identify predictors specifically associated with higher toxicity levels: (1) presence of oral mucositis, treated as a binary outcome (Grade 0 vs. Grades ≥1); (2) overall mucositis severity, defined as an ordinal outcome including the full range of grades (0, 1, 2, and 3); and (3) severity among affected patients, analyzed as an ordinal outcome restricted to individuals with documented mucositis (grades 1–3). These outcomes were analyzed separately to distinguish factors associated with the presence of mucositis from those related to its severity among affected patients.

### 2.5. Statistical analysis

Statistical analyses were performed using IBM SPSS Statistics version 25.0 (IBM Corp., Armonk, NY, USA). All tests were two‐tailed, and statistical significance was set at *p*  < 0.05.

For descriptive purposes, continuous variables were categorized using clinical criteria. Age was dichotomized as <60 or ≥60 years. RT sessions were grouped according to clinical timeframe (Early: 1–10; Intermediate: 11–20; Late: ≥21 sessions) and, among patients with RIOM (WHO grades 1–3), according to sample distribution (Early: ≤12; Intermediate: 13–17; Late: ≥18 sessions). Categorical variables were summarized using absolute and relative frequencies. Continuous variables were assessed for normality using the Kolmogorov–Smirnov test and described as mean ± standard deviation (SD) or median and interquartile range (IQR), as appropriate.

In bivariate analysis, Pearson’s chi‐square test or Fisher’s exact test was used for categorical variables. For ordinal predictors and binary outcomes, the chi‐square test for linear trend was applied when a monotonic trend was hypothesized. Comparisons of continuous variables across more than two outcome groups were performed using one‐way analysis of variance (ANOVA) or the Kruskal–Wallis test, according to distributional assumptions. Student’s *t* test or Mann–Whitney *U* test was used for two‐group comparisons. Spearman’s rank correlation coefficient was applied to assess associations between continuous RT exposure variables (number of sessions and cumulative dose) and ordinal RIOM severity.

Multivariable regression analyses were performed separately according to outcome scale. Binary logistic regression was used to evaluate factors associated with the presence of RIOM (WHO grade 0 vs grades ≥1). Ordinal logistic regression models with a logit link function were applied to assess factors associated with overall RIOM severity (WHO grades 0–3). Variables with *p*  < 0.20 in bivariate analyses or clinical relevance were included using the Enter method. All models were adjusted for age as a continuous variable, with sex, tumor location, and preventive laser therapy entered as categorical predictors. RT exposure was included as either total number of sessions or cumulative radiation dose; collinearity was addressed by fitting separate models for each exposure metric. Adjusted odds ratios (aORs) and 95% confidence intervals (95% CIs) were calculated.

Model fit for binary logistic regression was evaluated using the omnibus chi‐square test, Hosmer–Lemeshow goodness‐of‐fit test, receiver operating characteristic (ROC) curve analysis with area under the curve (AUC), and overall classification accuracy. For ordinal logistic regression, model fit was assessed using likelihood ratio chi‐square and pseudo‐*R* ^2^ measures (Cox & Snell and Nagelkerke), calibration using Pearson and deviance goodness‐of‐fit tests, and the proportional odds assumption using the test of parallel lines.

## 3. Results

A total of 151 records were screened, of which 30 were excluded due to concomitant chemotherapy (*n* = 22) and incomplete or missing data (*n* = 8). Consequently, data from 121 HNC patients were included in the final analysis.

According to dental records, all patients have undergone use of 3D conformal RT and underwent oral cavity preparation prior to antineoplastic treatment, including dental extractions and scaling and root planing when indicated. Preventive laser therapy was delivered according to a standardized protocol, consisting of low‐level red laser application three times per week (1 J, 3 s per point), with strict avoidance of the tumor area. All patients who developed RIOM received photobiomodulation therapy using low‐level red laser (1–5 J, 3 s per point) applied directly to ulcerated sites. Tumor staging and information on cancer‐related risk factors, such as tobacco and alcohol consumption, were inconsistently reported in the medical records and could not be reliably analyzed.

### 3.1. Participant Characteristics

The sample was predominantly male (66.9%) and aged ≥60 years (66.1%; range 9–96 years). Extraoral tumors were more frequent (58.7%), and squamous cell carcinoma accounted for most cases (91.7%). Preventive laser therapy was recorded in 55.4% of patients. The mean number of RT sessions was 21.0 ± 8.7, with a median of 19 sessions.

RIOM was identified in 51 patients (42.1%). Among affected patients, most were male (56.9%) and aged ≥60 years (60.8%). Intraoral tumors predominated (70.6%), and a lower proportion received preventive laser therapy (41.2%). Patients with RIOM had a higher median number of RT sessions (28), with 58.8% undergoing 21–35 sessions. At the time of RIOM diagnosis, the mean number of RT sessions was 15.1 ± 7.5, and the mean cumulative dose was 31.3 ± 15.1 Gy. WHO Grade 2 was most frequent (49.0%), and no cases of Grade 4 were observed.

### 3.2. Factors Associated With the Presence of RIOM

Bivariate analyses comparing patients without mucositis (WHO Grade 0) and those with mucositis (WHO Grades 1–3) are presented in Table 1. Patients with intraoral tumors presented a substantially higher prevalence of RIOM than those with extraoral tumors (70.6% vs. 20.0%, *p*  < 0.001), corresponding to an almost tenfold increase in odds (OR = 9.60; 95% CI: 4.14–22.24). Absence of preventive laser therapy was also associated with higher RIOM prevalence (58.8% vs. 41.2%, *p* = 0.007; OR = 2.74; 95% CI: 1.30–5.77).

**Table 1:** Sociodemographic, clinical, and radiotherapy‐related characteristics according to the presence of radiation‐induced oral mucositis (WHO Grade 0 vs. Grades 1–3) in patients with head and neck cancer undergoing radiotherapy (*n* = 121) in Sobral, Ceará, Brazil.

Variables	Total sample	No RIOM (grade 0)	RIOM present (grades 1–3)	*p*‐Value	OR (95% CI)
Sex, *n* (%)				0.44^a^	2.19 (1.01–4.74)
Male	81 (66.9)	52 (74.3)	29 (56.9)	—	—
Female	40 (33.1)	18 (25.7)	22 (43.1)	—	—
Age (years)	—	—	—	0.507^b^	—
Mean (SD)	62.8(16.1)	63.5 (15.5)	61.8 (17.2)	—	—
Max–Min	9–96	11–88	9–96	—	—
Median (IQR)	64(56–73)	65(56–74)	64(56–72)	—	—
Age group, *n* (%)				0.290^a^	0.66 (0.31–1.42)
<60 years	41 (33.9)	21 (30.0)	20 (39.2)	—	—
≥60 years	80 (66.1)	49 (70.0)	31 (60.8)	—	—
Tumor location, *n* (%)				<0.001^a^^∗^	9.60 (4.14–22.24)
Extraoral	71 (58.7)	56 (80.0)	15 (29.4)	—	—
Intraoral	50 (41.3)	14 (20.0)	36 (70.6)	—	—
Histological type, *n* (%)				1^a^	0.91 (0.24–3.40)
SCC	111 (91.7)	64 (91.4)	47 (92.2)	—	—
Other	10 (8.3)	6 (8.6)	4 (7.8)	—	—
Preventive laser therapy, *n* (%)	—	—	—	0.007^a^^∗^	2.74 (1.30–5.77)
Yes	67 (55.4)	46 (65.7)	21 (41.2)	—	—
No	54 (44.6)	24 (34.3)	30 (58.8)	—	—
Total RT Sessions (continuous)	—	—	—	<0.001^b^^∗^	—
Mean (SD)	21.0 (8.7)	18.6 (8.4)	24.4 (8.0)	—	—
Min–Max	3–35	3–35	5–35	—	—
Median (IQR)	19(15–29)	17(13–26)	28(18–31)	—	—
Total RT sessions (categorical), *n* (%)				0.007^c^^∗^	—
1–10 sessions	13 (10.7)	10 (14.3)	3 (5.9)	—	—
11–20 sessions	54 (44.6)	36 (51.4)	18 (35.3)	—	—
21–35 sessions	54 (44.6)	24 (34.3)	30 (58.8)	—	—
Accumulated dose (Gy)	—	—	—	0.001^b^^∗^	—
Mean (SD)	44.5(16.8)	40.2(16.7)	50.3(15.2)	—	—
Min–Max	8–70	8–70	10–70	—	—
Mediana	46(32–59)	40(30–52)	56(38–62)	—	—

*Note:* Statistical significance was set at *p*  < 0.05. ^∗^ means without statistical significance.

Abbreviations: CI, confidence interval; Gy, Gray; IQR, interquartile range; OR, odds ratio; RIOM, radiation‐induced oral mucositis; RT, radiotherapy; SCC, squamous cell carcinoma; SD, standard deviation; WHO, World Health Organization.

^a^Pearson’s chi‐square test or Fisher’s exact test for categorical variables.

^b^Mann–Whitney *U* test for continuous variables.

^c^For ordered categorical variables, the chi‐square test for linear trend was applied.

RT exposure was consistently greater among WHO Grades 1–3 patients. Both the number of sessions and the cumulative dose were significantly associated with RIOM. Patients who developed the condition received a higher median total dose (56 Gy vs. 40 Gy; *p* = 0.001) and completed more RT sessions (median: 28 vs. 17; *p* = 0.007). A significant dose–response trend was observed, with RIOM prevalence increasing from 5.9% between 1 and 10 RT sessions to 58.8% between 21 and 35 sessions (*p* = 0.007).

### 3.3. Overall Severity of RIOM (WHO Grades 0–3)

Analysis across the full spectrum of oral mucositis severity (WHO grades 0–3) demonstrated a clear gradient for RT exposure variables (Table 2).

**Table 2:** Radiotherapy exposure and clinical characteristics according to overall severity of radiation‐induced oral mucositis (WHO Grades 0–3) in patients with head and neck cancer undergoing radiotherapy (*n* = 121) in Sobral, Ceará, Brazil.

Variables	Grade 0 (*n* = 70)	Grade 1 (*n* = 13)	Grade 2 (*n* = 25)	Grade 3 (*n* = 13)	*p*‐Value	Spearman’s *ρ* (sig)
Sex, *n* (%)					0.102^b^	—
Male	52 (74.3)	6 (46.2)	16 (64.0)	7 (53.8)	—	—
Female	18 (25.7)	7 (53.8)	9 (36.0)	6 (46.2)	—	—
Age (years)					0.287^a^	−0.039 (*p* = 0.670)
Mean (SD)	63.5 (15.5)	61.9 (17.1)	58.7 (18.0)	67.6 (15.1)	—	—
Min–Max	11–88	30–82	9–96	39–92	—	—
Median(IQR)	65(56–74)	69(47–76)	60(55–67)	70(60–74)	—	—
Age group, *n* (%)	—	—	—	—	0.547^b^	—
<60 years	21 (30.0)	5 (38.5)	12 (48.0)	3 (23.1)	—	—
≥60 years	49 (70.0)	8 (61.5)	13 (52.0)	10 (76.9)	—	—
Tumor location, *n* (%)					<0.001^b^^∗^	—
Extraoral	56 (80.0)	3 (23.1)	9 (36.0)	3 (23.1)	—	—
Intraoral	14 (20.0)	10 (76.9)	16 (64.0)	10 (76.9)	—	—
Histological type, *n* (%)					0.464^b^	—
Squamous cell carcinoma (SCC)	64 (91.4)	10 (76.9)	16 (64.0)	10 (76.9)	—	—
Other histological types	6 (8.6)	3 (23.1)	9 (36.0)	3 (23.1)	—	—
Preventive laser therapy, *n* (%)					0.055^b^	—
Yes	46 (65.7)	3 (23.1)	12 (48.0)	6 (46.2)	—	—
No	24 (34.3)	10 (76.9)	13 (52.0)	7 (53.8)	—	—
Total RT sessions					0.002^a^^∗^	0.317 (*p* < 0.001)
Mean (SD)	18.6 (8.4)	25.9 (2.3)	25.0 (8.4)	27.0 (7.3)	—	—
Min–Max	3–35	5–34	9–35	12–35	—	—
Median (IQR)	17(13–26)	28(22–32)	25(16–31)	27(19–30)	—	—
Total RT sessions, *n* (%)					0.029^b^^∗^	—
1–10 sessions	10 (14.3)	1 (7.7)	2 (8.0)	0 (0.0)	—	—
11–20 sessions	36 (51.4)	2 (15.4)	10 (40.0)	6 (46.2)	—	—
21–35 sessions	24 (34.3)	10 (76.9)	13 (52.0)	7 (53.8)	—	—
Cumulative radiation dose (Gy)					0.011^a^^∗^	0.282 (*p* = 0.002)
Mean (SD)	40.2 (16.7)	51.9 (16.5)	48.8 (16.1)	51.7 (12.9)	—	—
Min–Max	8–70	10–68	18–70	32–70	—	—
Median(IQR)	40 (30–52)	56 (44–64)	52 (36–62)	55 (38–61)	—	—

*Note:* Spearman’s rank correlation coefficient (*ρ*) was used to assess monotonic associations between radiotherapy exposure variables and mucositis severity. Statistical significance was set at *p*  < 0.05.  ^∗^ means without statistical significance.

Abbreviations: Gy, Gray; RIOM, radiation‐induced oral mucositis; RT, radiotherapy; SCC, squamous cell carcinoma; SD, standard deviation; WHO, World Health Organization.

^a^Kruskal–Wallis test for continuous variables.

^b^The chi‐square test for linear trend for ordered categorical variables.

Higher mucositis grades were associated with a greater number of RT sessions (*p* = 0.002) and cumulative radiation dose (*p* = 0.011), with a moderate positive correlation between severity and number of RT session (Spearman’s *ρ* = 0.317, *p*  < 0.001) and between severity and cumulative dose (Spearman’s *ρ* = 0.282, *p* = 0.002).

Categorical analysis confirmed a linear trend, with a higher proportion of patients undergoing ≥21 RT sessions among grades 1–3 compared with grade 0 (*p* = 0.029). Tumor site remained strongly associated with severity (*p*  < 0.001), with intraoral tumors more frequent in higher grades. Preventive laser therapy showed a borderline inverse trend across severity grades (*p* = 0.055).

### 3.4. Factors Associated With Clinical Severity RIOM (WHO Grades 1–3)

Among patients with oral mucositis (WHO grades 1–3), no sociodemographic, clinical, or RT‐related factors were significantly associated with greater mucositis severity (Table 3). Cumulative radiation dose at the time of diagnosis did not differ across severity grades (*p* = 0.125), although a weak, non‐significant positive correlation with mucositis grade was observed (Spearman’s *ρ* = 0.225, *p* = 0.112).

**Table 3:** Bivariate analysis of sociodemographic, clinical, and radiotherapy‐related factors associated with clinical severity of radiation‐induced oral mucositis (WHO Grades 1–3) among affected patients with head and neck cancer undergoing radiotherapy (*n* = 51) in Sobral, Ceará, Brazil.

Variables	RIOM present (Grades 1–3)	Grade 1 (*n* = 13)	Grade 2 (*n* = 25)	Grade 3 (*n* = 13)	*p*‐Value	Spearman’s *ρ* (sig)
Sex, *n* (%)	—	—	—	—	0.695^b^	—
Male	29 (56.9)	6 (46.2)	16 (64.0)	7 (53.8)	—	—
Female	22 (43.1)	7 (53.8)	9 (36.0)	6 (46.2)	—	—
Age (years)	—	—	—	—	0.457^a^	0.106 (*p* = 0.457)
Mean (SD)	61.8 (17.2)	61.9 (17.1)	58.7 (18.0)	67.6 (15.1)	—	—
Min–Max	9–96	30–82	9–96	39–92	—	—
Median	64 (56–72)	69 (47–76)	60 (55–67)	70 (60–74)	—	—
Age group, *n* (%)	—	—	—	—	0.426^b^	—
<60 years	20 (39.2)	5 (38.5)	12 (48.0)	3 (23.1)	—	—
≥60 years	31 (60.8)	8 (61.5)	13 (52.0)	10 (76.9)	—	—
Tumor location, *n* (%)	—	—	—	—	1.000^b^	—
Extraoral	15 (29.4)	3 (23.1)	9 (36.0)	3 (23.1)	—	—
Intraoral	36 (70.6)	10 (76.9)	16 (64.0)	10 (76.9)	—	—
Histological type, *n* (%)	—	—	—	—	0.149^b^	—
SCC	47 (92.2)	10 (76.9)	16 (64.0)	10 (76.9)	—	—
Other	4 (7.8)	3 (23.1)	9 (36.0)	3 (23.1)	—	—
Preventive laser, *n* (%)	—	—	—	—	0.237^b^	—
Yes	21 (41.2)	3 (23.1)	12 (48.0)	6 (46.2)	—	—
No	30 (58.8)	10 (76.9)	13 (52.0)	7 (53.8)	—	—
Total RT sessions	—	—	—	—	0.723^a^	−0.059 (*p* = 0.681)
Mean (SD)	24.4 (8.0)	25.9 (2.3)	25.0 (8.4)	27.0 (7.3)	—	—
Min–Max	5–35	5–34	9–35	12–35	—	—
Median	28 (18–31)	28 (22–32)	25 (16–31)	27 (19–30)	—	—
Total RT sessions, *n* (%)	—	—	—	—	0.521^b^	—
1–10 sessions	3 (5.9)	1 (7.7)	2 (8.0)	0 (0.0)	—	—
11–20 sessions	18 (35.3)	2 (15.4)	10 (40.0)	6 (46.2)	—	—
21–35 sessions	30 (58.8)	10 (76.9)	13 (52.0)	7 (53.8)	—	—
RT sessions at RIOM diagnosis	—	—	—	—	0.181^a^	0.179 (*p* = 0.210)
Mean (SD)	15.1 (7.5)	12.8 (9.1)	16.3 (7.5)	15.1 (5.7)	—	—
Min–Max	3–34	3–31	3–34	7–27	—	—
Median	13 (10–18)	9 (6–20)	16 (10–18)	14 (11–18)	—	—
Total RT Sessions at RIOM diagnosis, *n* (%)	—	—	—	—	0.484^b^	—
≤12 (3–12) sessions	21 (41.2)	9(69.2)	7 (28)	5 (38.5)	—	—
13–17 sessions	15 (29.4)	0	10 (40)	5 (38.5)	—	—
≥18 (18–34) sessions	15 (29.4)	4(30.8)	8 (32)	3 (23)	—	—
Cumulative radiation dose at RIOM diagnosis (Gy)	—	—	—	—	0.125^a^	0.225 (*p* = 0.112)
Mean (SD)	31.3(15.1)	25.7 (18.2)	33.8 (15.0)	32.8 (11.1)	—	—
Min–Max	6–68	6–62	6–68	14–54	—	—
Median	28 (20–40)	18 (13–41)	32 (22–38)	30 (25–39)	—	—

*Note:* Analyses were restricted to patients presenting oral mucositis (WHO Grades 1–3). Spearman’s rank correlation coefficient (ρ) was applied where appropriate. Statistical significance was set at *p*  < 0.05.

Abbreviations: Gy, Gray; RIOM, radiation‐induced oral mucositis; RT, radiotherapy; SCC, squamous cell carcinoma; SD, standard deviation; WHO, World Health Organization.

^a^Kruskal–Wallis test for continuous variables.

^b^The chi‐square test for linear trend for ordered categorical variables.

### 3.5. Multivariable Analysis for the Presence of Oral Mucositis

In multivariable binary logistic regression analyses, tumor location and RT burden were the strongest independent predictors of oral mucositis occurrence (Table 4 and Figure 1A–C). In both adjusted models, intraoral tumor location was consistently associated with a markedly increased likelihood of mucositis when compared with extraoral tumors (Model A: aOR = 9.06; 95% CI: 3.51–23.38; *p*  < 0.001; Model B: aOR = 10.39; 95% CI: 3.95–27.32; *p*  < 0.001).

Figure 1Relationship between radiotherapy exposure and RIOM. (A) Scatter plot showing the distribution of mucositis presence (absent/present) across RT sessions; (B) Scatter plot of RIOM severity (WHO grades) as a function of cumulative RT sessions; (C) Predicted probability curve from binary logistic regression showing the increasing likelihood of RIOM as sessions progress; (D) Estimated probability of mucositis grades based on ordinal regression, highlighting the dose‐dependent progression of toxicity.

**Figure:**
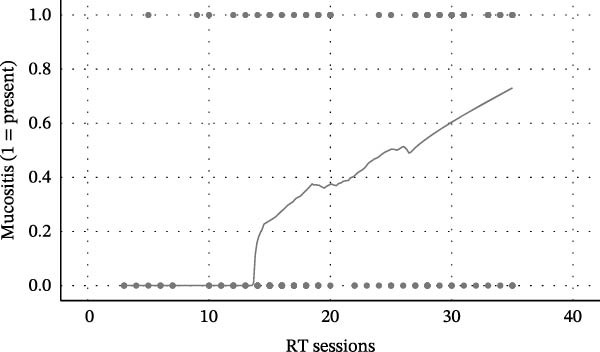
(A)

**Figure:**
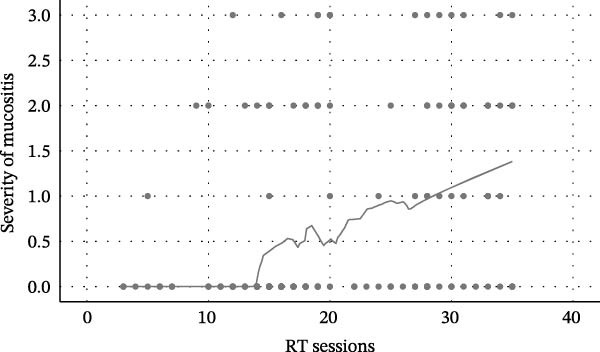
(B)

**Figure:**
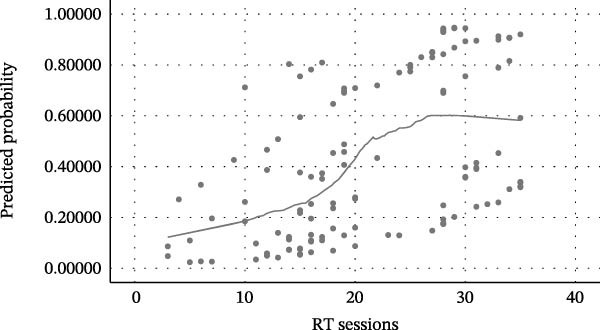
(C)

**Figure:**
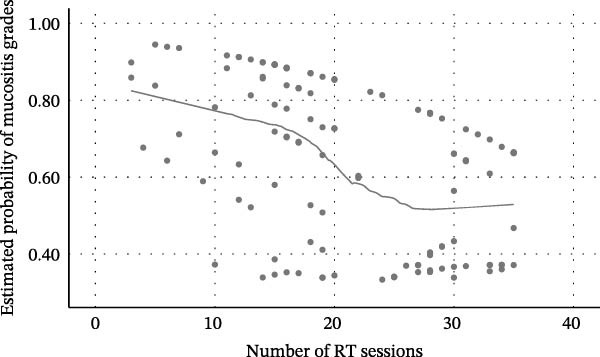
(D)

**Table 4:** Multivariable binary logistic regression analysis of factors associated with the presence of radiation‐induced oral mucositis (WHO Grade 0 vs. Grades 1–3) in patients with head and neck cancer undergoing radiotherapy (*n* = 121) in Sobral, Ceará, Brazil.

Predictor	Category/Unit	Model A aOR (95% CI)	*p*‐Value	Model B aOR (95% CI)	*p*‐Value
Sex	Male (reference)	—	—	—	—
	Female	3.097 (1.137–8.439)	0.027 ^∗^	2.651 (0.999–7.032)	0.050
Tumor location	Extraoral (reference)	—	—	—	—
	Intraoral	9.055 (3.507–23.381)	<0.001 ^∗^	10.391 (3.952–27.321)	< 0.001 ^∗^
Preventive laser therapy	Yes (reference)	—	—	—	—
	No	2.059 (0.803–5.279)	0.133	2.155 (0.836–5.551)	0.112
Age (years)	Continuous	0.994 (0.966–1.023)	0.705	0.994 (0.966–1.023)	0.681
Total RT sessions	Per additional session	1.109 (1.047–1.175)	<0.001 ^∗^	—	—
Total cumulative RT dose (Gy)	Per additional Gy	—	—	1.055 (1.024–1.088)	0.001 ^∗^

*Note:* The dependent variable was the presence of radiation‐induced oral mucositis, defined as WHO Grade 0 versus Grades 1–3. Model A included sex, age (continuous), tumor location, preventive laser therapy, and total number of radiotherapy sessions. Model B included sex, age (continuous), tumor location, preventive laser therapy, and cumulative radiation dose. Separate models were fitted to avoid collinearity between radiotherapy exposure variables. Statistical significance was set at *p*  < 0.05.  ^∗^ means without statistical significance.

Abbreviations: aOR, adjusted odds ratio; CI, confidence interval; Gy, Gray; RT, radiotherapy; WHO, World Health Organization.

RT exposure remained independently associated with mucositis. In Model A, each additional RT session was associated with an 11% increase in the odds of mucositis (aOR = 1.11; 95% CI: 1.04–1.17; *p*  < 0.001). In Model B, each 1 Gy increase in cumulative dose was associated with a 5% increase in mucositis odds (aOR = 1.05; 95% CI: 1.02–1.08; *p* = 0.001).

After adjusting for clinical and treatment factors, female patients showed significantly higher odds of presenting with RIOM in Model A (aOR = 3.1; 95% CI: 1.13–8.43; *p* = 0.027), with a similar borderline trend observed in Model B (*p* = 0.050).

Female sex was independently associated with mucositis in Model A (aOR = 3.10; 95% CI: 1.13–8.43; *p* = 0.027), with a borderline association in Model B (*p* = 0.050). Preventive laser therapy did not retain statistical significance after adjustment in either model, although a protective trend persisted. Age was not independently associated with the outcome.

Both models showed good fit and discrimination, with significant omnibus tests (*p*  < 0.001), adequate calibration, and excellent discriminative performance (AUC ≈ 0.85), with overall accuracy ranging from 76.9% to 77.7%.

### 3.6. Ordinal Regression Analysis of Oral Mucositis Severity (WHO Grades 0–3)

Consistent with the binary models, intraoral tumor location emerged as the strongest factor associated with greater oral mucositis severity (Table 5 and Figure 1D). In both adjusted ordinal models, intraoral tumors were strongly associated with higher mucositis grades compared with extraoral tumors (Model A: aOR = 5.99; 95% CI: 2.66–13.50; *p*  < 0.001; Model B: aOR = 6.56; 95% CI: 2.90–14.85; *p*  < 0.001).

**Table 5:** Multivariable ordinal logistic regression analysis of factors associated with overall severity of radiation‐induced oral mucositis (WHO Grades 0–3) in patients with head and neck cancer undergoing radiotherapy (*n* = 121) in Sobral, Ceará, Brazil.

Predictor	Category/Unit	Model A aOR (95% CI)	*p*‐Value	Model B aOR (95% CI)	*p*‐Value
Sex	Male (reference)	—	—	—	—
	Female	2.22 (0.99–5.00)	0.054	2.04 (0.92–4.52)	0.080
Tumor location	Extraoral (reference)	—	—	—	—
	Intraoral	5.99 (2.66–13.50)	<0.001 ^∗^	6.56 (2.90–14.85)	<0.001 ^∗^
Preventive laser therapy	Yes (reference)	—	—	—	—
	No	1.46 (0.66–3,25)	0.348	1.50 (0.67–3.33)	0.323
Age (years)	Continuous	0.99 (0.97–1.02)	0.955	0.99 (0.97–1.02)	0.945
Total RT sessions	Per additional session	1.07 (1.02–1.13)	0.003 ^∗^	—	—
Cumulative RT dose (Gy)	Per additional Gy	—	—	1.04 (1.01–1.06)	0.003 ^∗^

*Note:* The dependent variable was oral mucositis severity classified according to WHO Grades 0–3. Ordinal logistic regression models were fitted using a proportional odds assumption. Model A included sex, age (continuous), tumor location, preventive laser therapy, and total number of radiotherapy sessions. Model B included sex, age (continuous), tumor location, preventive laser therapy, and cumulative radiation dose. Separate models were estimated to address collinearity between radiotherapy exposure measures. Statistical significance was set at *p*  < 0.05.  ^∗^ means without statistical significance.

Abbreviations: aOR, adjusted odds ratio; CI, confidence interval; Gy, Gray; RT, radiotherapy; WHO, World Health Organization.

RT exposure remained independently associated with increasing severity. In Model A, each additional RT session was associated with a 7% increase in the odds of shifting to a higher mucositis grade (aOR = 1.07; 95% CI: 1.02–1.13). Similarly, in Model B, each 1 Gy increase in cumulative dose was associated with a 4% increase in the odds of higher severity (aOR = 1.04; 95% CI: 1.01–1.06). Both exposure metrics were statistically significant (*p* = 0.003).

Female sex showed a borderline association with higher severity in Model A (aOR = 2.22; 95% CI: 0.99–5.00; *p* = 0.054), whereas age and preventive laser therapy were not independently associated with mucositis severity in either model (*p*  > 0.30).

The ordinal regression models demonstrated adequate fit, with significant likelihood ratio tests (*p*  < 0.001), acceptable explanatory power (Nagelkerke *R* ^2^ ≈ 0.33), appropriate calibration, and fulfillment of the proportional odds assumption, supporting the validity of the ordinal logistic models. as indicated by non‐significant tests of parallel lines (Model 2A: *p* = 0.872; Model 2B: *p* = 0.921), supporting the validity of the ordinal logistic models.

## 4. Discussion

This study demonstrates that RIOM was a frequent complication among HNC patients undergoing 3D conformal RT and was strongly influenced by tumor location and RT burden. Intraoral tumors consistently showed a higher likelihood of RIOM occurrence and greater severity. RT exposure, expressed as either cumulative dose or number of sessions, exhibited a clear dose–response relationship with RIOM development. Preventive laser therapy showed a protective trend in unadjusted analyses but lost significance after adjustment. Sociodemographic factors had limited influence, and the early onset of RIOM suggests that RT characteristics and baseline clinical factors may affect the timing of oral toxicity.

RT burden demonstrated a consistent dose–response relationship with oral mucositis, supporting its role as a key determinant of both occurrence and severity. RIOM has been frequently described during the early phases of RT [12] and in this study, RIOM was documented across different RT phases, including a relevant proportion of cases recorded within the first 12 sessions, at a median cumulative dose of 28 Gy. Although some reports describe higher dose thresholds (30 Gy) in other settings [12], this finding may be related to the use of 3D conformal RT, which provides less sparing of the oral mucosa than intensity‐modulated RT [13]. Another possible reason for the early development RIOM is the large number of elderly patients since the healing and repair process is different from that of a younger patient (Table 1). Besides WHO classification (adopted in this study) may have been overestimated since even a small erythema could be considered RIOM grade 1.

The prevalence of RIOM observed in this sample (42.1%) was lower than that reported in several studies (80% or higher) [14, 15], which may reflect differences in RT protocols, assessment criteria, and preventive care. The WHO grading system was adopted in this study due to its widespread clinical use and its ability to capture both clinical signs and functional impairment. However, variability in grading systems and underreporting of mild presentations contribute to heterogeneity across epidemiological estimates [9].

Comprehensive dental care and oral environment stabilization prior to RT may also have contributed to the lower prevalence observed. Eliminating infectious foci and dental biofilm is known to mitigate the severity of the inflammatory response during RT [16]. Although preventive laser therapy showed a protective trend in unadjusted analyses, this association did not remain independent after adjustment, suggesting interaction with tumor location and RT burden.

Squamous cell carcinoma was the predominant histological type across most head and neck sites, which is consistent with the established epidemiology of these tumors [1]. Although women comprised a smaller proportion of the sample, female sex was associated with a higher likelihood of RIOM. This finding may reflect sex‐related differences in tissue sensitivity or inflammatory response to RT [17, 18], but should be interpreted cautiously, as behavioral factors were not assessed. Age was not independently associated with RIOM, suggesting that under high radiation burden and intraoral tumor involvement, the magnitude of radiation injury may outweigh age‐related differences in tissue repair capacity [15, 19].

This study has limitations inherent to its observational, cross‐sectional, and retrospective design, which precludes causal inference and longitudinal assessment of oral mucositis onset, progression, and late toxicities. Reliance on non‐standardized medical records may have introduced information bias. The single center setting and moderate sample size may limit external validity and statistical power for subgroup analyses. In addition, unmeasured or inconsistently recorded factors, including nutritional status, oral hygiene, alcohol use, smoking intensity, and genetic susceptibility, could not be accounted for, resulting in potential residual confounding.

## 5. Conclusion

In this Brazilian sample of patients with HNC treated with 3D conformal RT, RIOM was frequent. Intraoral tumor location and RT burden (number of sessions and cumulative dose) were the main factors associated with the presence and overall severity of mucositis (WHO Grades 0–3). Preventive laser therapy showed a protective trend in bivariate analysis but did not remain significant after adjustment. No variables were associated with increasing severity among affected patients (WHO grades 1–3). These findings underscore the importance of tumor site and radiation exposure for risk stratification and early preventive care during RT.

## Funding

No funding was received for this manuscript.

## Conflicts of Interest

The authors declare no conflicts of interest.

## Data Availability

The data that support the findings of this study are available on request from the corresponding author. The data are not publicly available due to privacy or ethical restrictions.
